# Circulating Tumor Cells in Gastrointestinal Cancers: Current Status and Future Perspectives

**DOI:** 10.3389/fonc.2019.01427

**Published:** 2019-12-13

**Authors:** Chaogang Yang, Fangfang Chen, Shuyi Wang, Bin Xiong

**Affiliations:** ^1^Department of Gastrointestinal Surgery, Zhongnan Hospital of Wuhan University, Wuhan, China; ^2^Hubei Key Laboratory of Tumor Biological Behaviors, Wuhan, China; ^3^Hubei Cancer Clinical Study Center, Wuhan, China; ^4^Department of Breast and Thyroid Surgery, Zhongnan Hospital of Wuhan University, Wuhan, China

**Keywords:** circulating tumor cells, gastric cancer, colorectal cancer, detection, identification, clinical application

## Abstract

Circulating tumor cells (CTCs), which are now defined as the “break away” cancer cells that derive from primary- or metastatic-tumor sites and present in the bloodstream, are considered to be the precursors of metastases. Considering the key role of CTCs in cancer progression, researchers are committed to analyze them in the past decades and many technologies have been proposed for achieving CTCs isolation and characterization with highly sensitivity and specificity until now. On this basis, clinicians gradually realize the clinical values of CTCs' detection through various clinical studies. As a “liquid biopsy,” CTCs' detection and measurement can supply important information for predicting patient's survival, monitoring of response/resistance, assessment of minimal residual disease, evaluating distant metastasis, and sometimes, customizing therapy choices. Nowadays, eliminating CTCs of the blood circulation has been regarded as a promising method to prevent tumor metastasis. However, research on CTCs still faces many challenges. Herein, we present an overview to discuss the current concept of CTCs, summarize the available techniques for CTCs detection, and provide an update on the clinical significance of CTCs in gastrointestinal malignancies, especially focus on gastric and colorectal cancer.

## Introduction

According to the GLOBOCAN 2018 reports, cancer is estimated to rank as the leading cause of death worldwide (1). Gastrointestinal (GI) malignancies, an important component of solid tumors, bear a heavier cancer-associated burden (2). At present, metastasis remains the main cause for GI malignancy-related deaths (3). Even for the early-stage patients who underwent curative resection, a considerable portion suffer metastatic disease within 5 years of surgery (4). This evidence implies that an occult metastatic process is parallel with primary tumor development (5) or that tumor cells with metastatic potential have entered the bloodstream from the primary tumor site during surgery and cause subsequent distant metastasis in the aforementioned patients (6). These cells are termed circulating tumor cells (CTCs), which have been proposed to be the important mediators of hematogenous metastasis of solid malignant tumors (6, 7).

CTCs, first reported by Ashworth in 1869 and further demonstrated by Engell in 1955, are now defined as the “break away” cancer cells that derive from primary or metastatic tumor sites and present in the blood circulation (8). These cells shed intermittently from the tumor site, circulate within the bloodstream, potentially seed into distant organs and finally form vital metastases (8). Therefore, research on CTCs can provide more insights into metastasis-associated progression. However, the extremely low concentration in the peripheral blood (one CTC in millions of blood cells) makes CTCs detection a technical challenge (9), which in turn greatly limits in-depth studies on the biological properties of CTCs (10). Nevertheless, given the critical role of CTCs in tumor progression, many researchers have expended much effort to explore efficiently capture CTCs (9). Consequently, a considerable amount of scientific literature has published over the past decade, occurring in parallel with technical progress that has propelled this field forward. To date, a number of technologies based on the biological or physical properties of CTCs have been developed for achieving CTCs isolation and identification (9, 11–13), which lay the technical foundation for conducting more clinical research to explore the clinical value of CTCs detection in predicting patient survival, customizing therapy choices, monitoring response/resistance, and evaluating distant metastasis in numerous types of cancer (14). Over the past few years, our group has been working on CTCs detection methods and has developed a variety of methods based on the different biophysical characteristics of CTCs (15–24); these studies have enabled us to efficiently capture CTCs in the peripheral blood and to further analyze the prognostic value of quantitative and qualitative CTCs analysis in gastrointestinal (GI) malignancies (25–27).

In this review, we aim to outline the current status of CTCs detection techniques, the clinical implications, and the limitations and opportunities in GI cancers, including gastric cancer (GC) and colorectal cancer (CRC); we then provide new insights into the applications of CTCs detection to guide clinical practice.

## Isolation and Enrichment Technologies of CTCs

**Figure 1:**
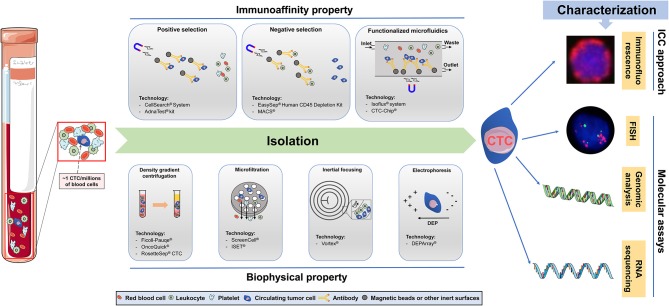
Overview of technologies for circulating tumor cells (CTCs) capture, enrichment, and characterization. Immunoaffinity-based enrichment technologies capture CTC by positive or negative selection, typically using antibodies bound to the device surface or to magnetic beads. Positive selection is based on the specific targeting of CTCs epithelial biomarkers, whereas negative selection depletes hematopoietic cells by targeting cell-surface antigens not expressed in CTCs. Functionalized microfluidics platforms can combine the advantages of microfluidic and the characters of positive capture and negative enrichment. Biophysical methods are label-free technologies relying on cell size, shape, density, and electric charge differences between CTC and other blood constituents. Density gradient centrifugation relies in the separation of different cell populations based on their relative densities. Microfiltration consists on size-based cell separation using pores or three-dimensional geometries. Inertial focusing relies on the passive separation of cells by size, through the application of inertial forces that affect positioning within the flow channel in microfluidics devices. Electrophoresis separates cells based on their electrical signatures, using an electric field. The methods of CTCs characterization include immunocytochemistry (ICC)-based approaches and molecular assays. Of which, ICC-based approaches are consist of immunofluorescence and immunohistochemistry technology, and molecular assays are consist of fluorescent *in situ* hybridization (FISH), real-time polymerase chain reaction (RT-PCR), genomic analysis, and RNA sequencing.

Although the primary tumor or metastasis site releases tumor cells into the blood at all times, most of them are eliminated by the body's immune system, and only a few CTCs survives in the blood circulation. Therefore, the number of CTCs is sparse (~1 CTC per ml of blood) compared to the number of other cellular components in the peripheral blood (5). This situation poses a high technical challenge for us to accurately isolate CTCs from millions of blood cells, indicating that an ideal technology for CTCs separation needs to have the following characteristics: (1) the ability to isolate all heterogeneous CTCs; (2) the ability to exclude the background interference caused by normal blood cells; and (3) the ability to accurately identify all candidate CTCs. At present, it has been well-recognized that the biological and physical characteristics of CTCs are obviously different from those of other cells in the blood (8). Consequently, many capture and identification technologies based on different CTCs features are gradually being developed to pursue the ultimate goal of achieving CTCs enrichment with high specificity and sensitivity (9, 11–13). For CTC enrichment, the isolation of CTCs is usually the first step, and the characterization of CTCs (the second step) further distinguishes the CTCs from the remaining normal blood cells. As shown in Figure 1, we presented an overview of the technologies utilized for CTCs isolation and characterization, and these technologies are commonly used in GC and CRC.

### Immunoaffinity-Based Technologies of CTCs

Immunoaffinity-based technologies, including positive or negative selection assays, achieve CTCs isolation with an antibody-immobilized inert surface combined with magnetic beads (28). Among these assays, positive selection assays frequently rely on two types of antigens, either single or a combination, that include the epithelial- or tumor-specific cell surface antigens (12). In the process of GC- and CRC-CTCs isolation, the most commonly used epithelial-specific cell surface antigens are cytokeratins (CKs) 18, 19, 20 and epithelial cell adhesion molecules (EpCAMs). CKs are intermediate filament keratins found in the cytoskeletons of epithelial cells (29). EpCAM is a human cell surface glycoprotein involved in cell-to-cell adhesion, which overexpresses in epithelial cancers and has been extensively used in proof-of-concept studies (30). Among tumor-specific cell surface antigens, carcinoembryonic antigen (CEA) has been largely utilized to isolate CRC-CTCs (31), and human epithelial growth factor receptor-2 (HER-2) was used for GC-CTCs isolation (32). Currently, several platforms, such as the CellSearch^®^ System and AdnaTest^®^ kit, have been developed for GC- and CRC-CTCs detection based on positive selection, and are now have achieved for commercially available (27, 33). Conversely, negative selection assays generally remove white blood cells (WBCs) from blood samples by targeting leukocyte surface-specific antigens (e.g., CD45 and CD61) that are not expressed in CTCs to achieve GC- and CRC-CTCs enrichment; the kits and techniques include the EasySep^®^ Human CD45 Depletion Kit (34) and MACS^®^ (35). Notably, Nagrath et al. developed the “CTC-Chip” platform by combining microfluidic technology with positive selection methods 10 years ago, and this method was able to selectively and efficiently isolate CTCs from whole blood using anti-EpCAM-coated posts with this microfluidic chip (36). Microfluidic devices are promising technologies for CTC isolation, which allow the separation of CTCs from small fluid volumes under laminar flow and eliminate the need for pre-labeling or sample processing (32). The Isoflux^®^ System (Fluxion Biosciences Inc., South San Francisco, CA) was another classic automated EpCAM-based immunoaffinity functionalized microfluidic system that used immunomagnetic beads to facilitate the use of single or multiple capture antibodies to target cells of a specific pathology, providing near-perfect isolation efficiency (37). Although, given that there are no 100% tumor-specific antibodies, the false-positive (specificity) and false-negative (sensitivity) of CTCs isolation continue to impose shackles on immuno-magnetic detection techniques.

Among the commercially available semiautomated devices, the CellSearch^®^ System (Veridex LLC, Raritan, NJ, USA) is the most reported immunoaffinity (EpCAM-based) method for CTCs isolation and counting, which has been approved by the Federal Drug And Food Administration (FDA) for use in metastatic breast and colon cancer patients (38). Additionally, it has also been widely used in the capture of GC and CRC-CTCs in recent years (27, 31). As one of the immunoaffinity assays, the major advantages of the CellSearch^®^ System are the direct visualization and quantification of CTCs and the detection of living cells without the need for cell lysis. However, there is a non-negligible fact that CellSearch detects a relatively low number of CTCs from the peripheral blood of patients with cancer, and this low sensitivity may be because the system captures solely EpCAM-positive CTCs that are significantly reduced or absent in certain CTCs subpopulations, especially for those undergoing epithelial-to-mesenchymal transition (EMT); this characteristic is still considered a major pitfall of this device (38).

Previously, our group also reported several immunoaffinity-based technologies for CTCs detection. First, we developed a new CTCs detection platform by using an electrospun TiO2 nanofiber-deposited substrate grafted with anti-EpCAM, which achieved high efficiency in CTCs detection from the blood of GC and CRC patients (15). Meanwhile, a new CTCs capture platform based on the transparent and biocompatible TiO2 nanoparticle spin coated on a glass substrate conjugated with anti-EpCAM also was successfully used to capture GC- and CRC-CTCs (16, 17). However, preparation of the above nanostructures requires either specialized equipment or complex process control, which limits its high-throughput fabrication. Moreover, the non-transparent nature makes them incompatible with many optical imaging systems (such as immunocytochemical techniques), which also constrains further application. Therefore, our group further used a hydroxyapatite/chitosan (HA/CTS) material as a nano-substrate, which was characterized by transparency and excellent biological compatibility, and conjugated this material with anti-EpCAM to develop simple but efficient CTCs detection platforms (18, 22). More importantly, the enumeration of CTCs by these platforms in GC patients could predict the clinical response to anticancer therapy (19). Furthermore, we coated anti-CD45 and anti-EpCAM onto the surface of the above nano-substrate to develop a combined negative and positive enrichment assay, exhibiting equally high capture efficiency and excellent purity for CRC-CTCs detection (21).

### Biophysical Property-Based Technologies of CTCs

Considering the bias and narrow capture spectrum presented by the aforementioned immunoaffinity-based approaches in CTCs isolation, researchers began to develop a variety of CTCs isolation technologies based on the biophysical properties of CTCs to achieve a wide-scale and high-performance capture of CTCs (39). Biophysical CTCs enrichment technologies, characterized as “label-free,” isolate CTCs from the blood based on the biophysical property differences, such as density, size, deformability, and electrical charge, that present among CTCs and other blood cells for CTCs separation and capture (40). Recently, there have been commercially available reagents and platforms based on the above different principles for separating GC- and CRC-CTCs, including density gradient centrifugation (Ficoll-Pauqe^®^ OncoQuick^®^ RosetteSep^®^ CTC), microfiltration (ScreenCell^®^ ISET^®^), inertial focusing (Vortex^®^), and electrophoresis (DEPArray^®^) (41). The most common biophysical CTCs enrichment technology is size-based microfiltration, which assumes that CTCs can be isolated from blood cells due to their larger volume and more rigid shape, and this technology has been improved by the introduction of nano to micron-sized filter pores (42). Currently, new lab-on-a-chip microfluidics devices have gradually appeared and significantly improved the GC- and CRC-CTCs yields compared with the conventional membrane microfiltration and EpCAM-based immunoaffinity assays (43, 44). Moreover, these technologies have provided improved *in situ* platforms for molecular analysis by fluorescent *in situ* hybridization (FISH) or immunofluorescence (IF) (45), as well as for the extraction of biomolecules for downstream genomic and transcriptomic sequencing (43). In addition, these platforms also provide the opportunity for CTCs release and *ex vivo* expansion, which lays an important foundation to further understand the biological characteristics of CTCs (46).

Previously, our group reported several biophysical property-based assays of CTCs detection. We fabricated a label-free wedge-shaped microfluidic chip (named CTC-Δchip) based on the size characteristics of CTCs, which exhibited high performance in capturing GC-CTCs and a great potential clinical value (24). Additionally, our group co-operated with YZY Medical Science and Technology Company (Wuhan, China) to develop a novel isolation by size of epithelial tumor cells device named CTCBIOPSY^®^ (Wuhan YZY Medical Science and Technology Co., Ltd., Wuhan, China), which achieved CTCs isolation and identification through a polymer membrane made by biocompatible parylene and Wright's staining (23). As a one-stop ISET device, CTCBIOPSY^®^ exhibited excellent performance in capturing patients' CTCs and has now been approved by the China Food and Drug Administration (CFDA) for clinical application in cancer management (23, 26).

### Molecular (RNA-Based) Assays of CTCs (Without Prior Enrichment)

The aforementioned immunoaffinity- or biophysical property-based technologies of CTCs detection need to separate GC- and CRC-CTCs from blood cells before identification. Molecular assays, represented by RT-PCR, can directly achieve the detection and characterization of CTCs by analyzing the expression of GC and CRC-CTCs-related genes without prior CTCs enrichment (47, 48). In contrast to enrichment technologies, RT-PCR has the advantages of being rapid, well-implemented, sensitive, and cost effective (41). Previously, our group conducted a series of meta-analyses to explore the clinical role of CTCs detected by RT-PCR in GC and CRC and summarized the commonly used markers for GC-CTCs (including CK19, CK20, CEA, hTerT, c-MET, MUC1, VEGFR-1, Survivin, uPAR, B7-H3, and STCs) and CRC-CTCs (including CK19, CK20, CEA, PLS3, CD133, hTerT, EphB4, LAMγ2, and MAT) detection (25, 49). Using these cancer-related genes for CTCs detection is of great value in evaluating the prognosis of patients with both GC and CRC (25, 49). However, tumor-derived circulating RNAs (such as miRNAs and lncRNAs) present in the blood of cancer patients may affect the accuracy of RT-PCR for CTCs detection, contributing a major limitation of this technology (41).

### Molecular Characterizing Technologies of CTCs

After enrichment by the above platforms, the candidate CTCs need to be further identified as “true” CTCs. Currently, the identification and molecular characterization of CTCs is achieved by (a) immunocytochemistry (ICC)-based assays, including IF and immunohistochemistry (IHC), and (b) molecular approaches, including RT-qPCR, FISH and next-generation sequencing (NGS) (41). The most commonly used assay for the identification of GC- and CRC-CTCs from contaminating cells is IF, which achieves CTCs identification by staining and visualizing related-antibody biomarkers. Such biomarkers can be specific for nuclear content, epithelial proteins (i.e., CKs), mesenchymal proteins (i.e., vimentin), and hematopoietic markers (i.e., CD45). A common immunocytological CTC definition is nucleus+/CK+/vimentin–/CD45– cell for epithelial-CTC, nucleus+/CK–/vimentin+/CD45– cell for mesenchymal-CTC, and nucleus+/CK+/vimentin+/CD45– for epithelial/mesenchymal-CTC (50). However, the detection of CTCs by classical IF, which is typically performed by pathologists through the visual observation of stained CTCs based on the above principles, is time consuming and subjective-dependent. By contrast, PCR-based molecular assays provide objective and quantifiable CTCs measurements with the advantages of automated, sensitive, relatively low-cost and amenable to quantifiable quality control. Moreover, these methods require a small amount of cells for analysis, which is also in line with the fact that the amount of CTCs is less (51). However, since the molecular characterization of CTCs by PCR assays is based on the detection of mRNA markers that are specifically expressed in CTCs but not in leukocytes, the risk of false-positive results might be increased due to the non-specific amplification of RNA (50–52).

Notably, nucleic acid-based technologies, as improvements to non-fixating enrichment procedures, allow the use of RT-PCR and qRT-PCR to amplify single or multiple gene transcripts for CTCs detection, and these technologies have provided an alternate avenue for the molecular characterization of GC- and CRC-CTCs (53–55). In particular, recent emerging single-cell sequencing techniques, including DNA and RNA sequencing, have turned the research direction toward analyzing the genetic characteristics of individual CTCs to assist in exploring tumor metastasis mechanisms, finding drug targets, monitoring therapy responses, and assessing drug resistance (54). Although, because single-cell CTC analyses are limited by the heterogeneity between cancer subtypes, the usefulness of these analyses has hindered the discovery of universal markers (54).

## Clinical Value of CTCs Detection in Gastrointestinal Cancer

**Figure 2:**
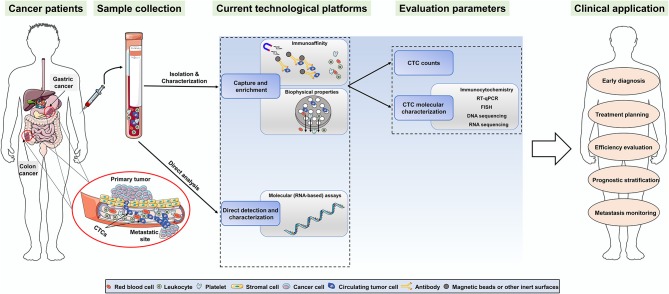
Overview of the CTCs detection technologies and the potential clinical applications of CTCs in gastric and colorectal cancer.

In recent years, the clinical applications of CTCs detection via various technologies have been gradually involved in multiple aspects of GI cancers, including early diagnosis, treatment planning, efficiency evaluation, prognostic stratification, and metastasis monitoring (56) (summarized in Figure 2). Despite this, there is still no universally applicable “gold standard” method so far (41, 56). Therefore, the aforementioned assays must be validated in clinical trials to achieve clinical validity and utility in the future.

### Prognostic Stratification

The role of CTCs in the prognostic stratification of patients with GC and CRC, as the most studied aspect of CTCs' clinical value, has been demonstrated by numerous studies (26, 57–91). For both GC and CRC, CTCs detection is considered to be significantly correlated with disease progression and patient's prognosis (56). Previously, our group conducted a prospective cohort study that recruited 138 patients with stage I–III CRC to assess the prognostic value of the change in CTCs counts before and after curative surgery. The results found that postoperative CTCs-positive but not preoperative CTCs-positive is an independent indicator of poor prognosis for CRC patients, and the patients with preoperative CTCs-positive that normalized after surgery have similar outcomes to patients with preoperative CTC-negative (26). Meanwhile, our clinical study demonstrated that combining the preoperative controlling nutritional status score and circulating tumor cell status could strongly predict the prognosis for CRC patients treated with curative resection (92), which indicated that the state of CTCs in the blood is closely related to the nutrition and immune status of the host. In addition, a series of meta-analyses conducted by our group also provided strong evidence for the prognostic significance of CTCs detection in GI malignancies, which showed that CTCs-positive predicts a poor patient prognosis and unfavorable clinicopathological factors for both GC and CRC, regardless of whether the detection method was RT-PCR, CellSearch or cytological methods (25, 27, 49, 93). In these processes, an unneglectable fact is that CTCs detection at different time points during treatment might exhibit different prognostic significance (14). The reason is that a cancer (or a minimal residual disease) evolves with time, treatment, selection pressure from surgery, chemotherapy and radiotherapy and that tumoricidal immunity could stimulate the expansion of tumor subclones, leading to a change in the number and molecular characteristics of CTCs (94). In the future, repeated CTCs detection may be necessary to capture the changing genetics attributed to anticancer therapies. In the present review, we summarized the prognostic value of CTCs detection using different methods at different time points in GC and CRC (summarized in Table 1). As shown in Table 1, although there are many CTCs detection methods, none of them are generally accepted and could be really applied to clinical practice. At the same time, the cut-off values of the same CTCs detection method are different from study to study. Therefore, it is necessary for larger clinical studies to further validate whether CTCs are used in clinical practice to guide prognostic assessment. Of course, this may still have a long way to go.

**Table 1:** CTCs detection for prognosis of gastric and colorectal cancer.

**Cancer types**	**Cut-off value**	**Technique**	**Patients (*n*)**	**HR for death (95% CI)**		**HR for progression/recurrence (95% CI)**		**References**
				**Before treatment**	**After treatment**	**Before treatment**	**After treatment**	
Gastric cancer	≥2.8 CTCs	ISET	Non-metastatic GAC (88)	–	–	–	–	(57)
	≥5 CTCs	CellSearch^®^	Resectable GC (93)	–	–	–	–	(67)
	>17 CTCs	IsoFlux^®^	Stage II–IV EGC (43)	3.7 (1.2–12.4)	–	–	–	(58)
	≥1 CTCs	ISET	Stage II–IV GC (86)	2.96 (1.25–7.04)	-	3.94 (1.38–11.27)	–	(68)
	>2 CTCs	CELLection™	Stage II–IV GC (59)	3.59 (1.66–7.82)	0.77 (0.27–2.25)	2.81 (1.31–6.00)	6.58 (1.37–31.6)	(63)
	≥4 CTCs	SE-iFISH	Advanced GC (31)	–	–	–	–	(62)
	>5 CTCs	GFP fluorescence	Stage II–IV GC (65)	0.90 (0.29–2.59)	–	1.97 (0.47–8.86)	–	(59)
	≥3 CTCs	CellSearch^®^	Advanced EGC (106)	–	3.46 (1.82–6.58)	–	2.15 (1.11–4.16)	(61)
	≥2 CTCs	Cytometry, FISH	Advanced EGC (60)	4.30 (0.82–22.90)	–	6.70 (1.43–31.03)	–	(64)
	≥1 CTCs	CellSearch^®^	Advanced GC (136)	1.37 (0.68–2.77)	–	2.14 (1.09–4.20)	–	(65)
	≥5 CTCs	CellSearch^®^	Advanced GC (100)	2.58 (1.57–4.27)	–	2.06 (1.26–3.38)	–	(60)
	≥1 CTCs	CellSearch^®^	Resectable GC (148)	1.73 (1.08–2.77)	–	–	–	(66)
Colorectal cancer	≥3 CTCs	Cyttel+imFISH	Advanced CRC (121)	–	2.68 (1.19-6.03)	–	2.79 (1.01–7.71)	(69)
	≥4 CTCs	CellSearch^®^	Non-metastatic CRC (63)	41.03 (0.00–102.40)	–	17.6 (3.7–82.6)	–	(70)
	≥1 CTCs	ISET	Non-metastatic CRC (138)	–	–	2.17 (0.75–6.31)	2.82 (1.39–5.75)	(26)
	≥1 CTCs	Immunomagnetic selection	mCRC (77)	0.32 (0.72–2.79)	0.35 (0.12-0.99)	–	–	(71)
	≥1.92 CTCs	CEACAM5 RT-PCR	mCRC (436)	2.1 (1.3–3.2)	–	1.6 (1.1–2.5)	–	(72)
	≥6 CTCs	CanPatrol™	Stage I-IV (66)	59.7 (0.002–1.6 ×10^6^)	–	7.42 (1.06–51.74)	–	(73)
	>30 CTCs	Vita-Assay™	Stage I-IV (88)	1.04 (1.01–1.06)	–	–	–	(74)
	≥2 CTCs	CellSearch^®^	mCRC (79)	2.51 (0.69–9.09)	–	3.28 (1.24–8.67)	–	(75)
	>30 CTCs	Negative selection	mCRC (55)	2.61 (1.39–4.93)	–	4.94 (2.60–9.39)	–	(76)
	NR	Multiparameter flow cytometry	mCRC (152)	6.46 (1.46–28.56)	–	–	–	(77)
	≥1 CTCs	ISET	Stage II-IV (98)	–	1.15 (0.68-1.94)	–	1.99 (1.14–3.48)	(78)
	≥1+ PCR test out of 3	CK20 RT-PCR	Resectable colon cancer (299)	1.94 (1.0–3.7)	–	1.94 (1.1–3.7)	–	(79)
	≥1 CTC	CellSearch^®^	Stage I–III CRC (239)	5.5 (2.3–13.6)	–	12.7 (5.2–31.1)	–	(80)
			Stage I–IV CRC (287)	5.6 (2.6–12.0)	–	7.8 (3.9–15.5)	–	
	≥1 CTC	CellSearch^®^	Stage III CRC (519)	–	0.96 (0.56-1.65)	–	0.97 (0.65–1.45)	(81)
	≥2 CTCs	CellSearch^®^	Resectable CRC LM (194)	2.48 (1.40–4.38)	–	2.32 (1.26–4.27)	–	(82)
	>0.1 ng/μL for ≥1 out of 3 gene	AdnaTest^®^	Metastatic RAS-BRAF wt CRC (38)	9.32 (2.63–33.1)	–	6.24 (2.54–15.3)	–	(83)
	≥1 CTC	CellSearch^®^	Resectable colon cancer (183)	2.88 (1.46–5.66)	–	1.96 (1.06–3.61)	–	(84)
	≥3 CTCs	CellSearch^®^	Metastatic KRAS wt CRC (63)	2.08 (1.16–3.73)	–	–	–	(85)
	≥1 CTC	CellSearch^®^	mCRC (119)	–	–	2.05 (1.29–3.28)	–	(86)
	≥3 CTCs	CellSearch^®^	mCRC (180)	1.54 (1.00–2.37)	–	1.47 (0.98–2.22)	–	(87)
	≥3 CTCs	CellSearch^®^	mCRC (64)	–	1.44 (1.14–1.82)	1.06 (0.98–1.15)	1.21 (1.09–1.34)	(88)
	All markers positive	CK19, CK20, CEA, CD133 RT-PCR	Resectable CRC (315)	3.20 (1.67–6.31)	–	3.04 (1.79–5.22)	–	(89)
	≥3 CTCs	CellSearch^®^	mCRC (467)	1.9	–	1.4	–	(90)
	>3 CTCs	CellSearch^®^	mCRC (430)	2.45 (1.77–3.39)	9.35 (5.28–16.54)	1.74 (1.33–2.26)	3.64 (2.10–6.30)	(91)

*CTCs, circulating tumor cells; HR, hazard ratio; CI, confidence interval; ISET, isolation by size of epithelial tumor cells; GAC, gastric adenocarcinoma; GC, gastric cancer; EGC, esophagogastric cancer; FISH, fluorescent in situ hybridization; CRC, colorectal cancer; mCRC, metastatic CRC; RT-PCR, real-time polymerase chain reaction; CK, cytokine; CEA, carcinoembryonic antigen; wt: wild type; LM, lung metastasis; NR, not reported*.

### Therapeutic Implications

Currently, there is limited evidence showing that CTCs detection at baseline can predict the response to systemic therapy in GI cancers (60, 61, 83, 85, 87, 88, 90, 91, 95–98). However, a few studies have demonstrated the predictive value of CTCs detection during chemotherapy (summarized in Table 2). Li et al. conducted a single-center, prospective study to measure the level of CTCs before and at 6 weeks of chemotherapy in 136 patients with newly diagnosed advanced GC, and the results showed that the posttherapy CTCs levels may help evaluate the therapeutic response; in addition, the changes in CTCs following therapy may be useful in rapidly identifying ineffective treatments for patients with advanced GC (61). Similarly, a study including 430 patients with metastatic CRC also found that there were significantly higher disease progression rates among patients who were CTCs-positive after 3–4 weeks of chemotherapy (91). Additionally, CTCs have also been used as a vehicle to assess genotyping changes in primary tumor and metastatic lesions; this is relevant for patients for whom a targeted therapy against known resistance-causing mutations is available, such as HER2-directed treatment for GC and EGFR-directed treatment for CRC (14). Overall, although the therapeutic predictive value of CTCs is not as well-studied as their prognostic value, using CTCs detection for determining the choice of systemic treatment and monitoring the treatment effects is promising, illustrating the possibility of liquid biopsy assessments to change future cancer management.

**Table 2:** CTCs as predictive factors for cancer therapy efficacy in gastric and colorectal cancer.

**Cancer types**	**Cut-off value**	**Technique**	**Patients (*n*)**	**Treatment**	**Conclusions**	**References**
Gastric cancer	≥1 CTC	3D-IF-FISH method	Unresectable metastatic or recurrent GC (15)	1st-line CT + trastuzumab	ORR was 53.3% in CTCs-HER2 positive patients at first response evaluation (6 weeks) vs. 7.7% in CTCs-HER2 negative patients (*p* = 0.016)	(98)
	≥3 CTCs	CellSearch^®^	Advanced GC (106)	1st-line CT	ORR was 30.0% in CTCs-negative patients at first response evaluation	(61)
	≥5 CTCs	CellSearch^®^	Metastatic GC (100)	≥1st-line CT	Chemotherapy response (CR or PR or SD) was 76.6% in CTCs-negative patients vs. 40.0% in CTCs-positive patients (*p* = 0.004)	(60)
	≥1 of the marker genes positive	EpCAM + RT-PCR	Advanced GC (61)	1st or 2nd-line CT	100% of progressive patients were CTCs-positive at baseline vs. 73.5% of non-progressive patients (*p* = 0.003)	(96)
Colorectal cancer	2 + PCR results	RT-PCR	LARC (79)	CRT + surgery	After CRT, CTCs were detected in 54.4% of the non-responders vs. 27.2% of the responders (*p* = 0.030)	(95)
	≥1 CTC	CellSearch^®^	LARC (85)	CRT + surgery	pCR/downstaging/downsizing rate was 80% in baseline CTCs-negative patients vs. 40% in CTCs-positive patients (*p* = 0.02)	(97)
	≥1 out of 3 CTCs markers	AdnaTest^®^	Metastatic RAS-BRAF wt CRC (38)	≥1st-line CT	ORR in unfavorable and favorable CTCs-changes profiles were respectively 0% and 59% (*p* <0.0001)	(83)
	≥3 CTCs	CellSearch^®^	Metastatic KRAS wt CRC (61)	3rd-line CT	ORR was not different between the high and the low CTCs groups (27.7 vs. 18.36%, *p* = 0.498)	(85)
	≥3 CTCs	CellSearch^®^	mCRC (180)	1st-line CT	CTCs negativity after 3 cycles of CT was associated with higher ORR (OR, 3.22; 95% CI 1.25–9.43)	(87)
	≥3 CTCs	CellSearch^®^	mCRC (60)	1st or 2nd-line CT	CTCs positivity at 8–12 weeks was 2% in non-PD patients vs. 43% in PD patients (*p* = 0.004)	(88)
	≥3 CTCs	CellSearch^®^	mCRC (307)	1st-line CT	ORR was 40% in patients with low CTCs count at 1–2 weeks vs. 11% in patients with high CTCs count (*p* = 0.022)	(90)
	≥3 CTCs	CellSearch^®^	mCRC (430)	1st, 2nd, or 3rd CT	CTCs positivity at 3–5 weeks was 7% in non-PD patients vs. 27% in PD patients	(91)

*CTCs, circulating tumor cells; GC, gastric cancer; CRC, colorectal cancer; mCRC, metastatic CRC; LARC, localize advanced rectum cancer; IF, immunofluorescence; FISH, fluorescent in situ hybridization; EpCAM, epithelial marker epithelial cell adhesion molecule; HER2, human epidermal growth factor receptor 2; RT-PCR, real-time polymerase chain reaction; CT, chemotherapy; CRT, chemoradiotherapy; CR, complete response; PR, part response; SD, stable disease; ORR, overall response rate = complete response + partial response; OR, odds ratio*.

### Early Diagnosis

In the early stage of the disease, tumor cells may separate from the primary tumor and enter the bloodstream; this circumstance provides a theoretical basis for CTCs detection as a tool for early diagnosis. Over the past few years, several studies have explored the early diagnostic value of CTCs detection based on different methods in GI malignancies, and the results found that the fraction of patients positive for CTCs is generally considered too low to obtain sufficient sensitivities for true early diagnosis (66, 99–101). Therefore, screening general populations with a CTCs assessment is not logistically realistic, but may be realistic in the high-risk groups, such as those with a family history of GI cancers.

## Concluding Remarks

Although the detection and measurement of CTCs is expected to become a promising tool as prognostic, predictive, and diagnostic markers for patients with GC and CRC, CTCs have yet to be realized owing to residual surmountable challenges. To achieve this goal, a CTCs detection device that is universally accepted, fast, and low-cost with low false-negative and false-positive results is first needed; simultaneously, standard procedures for CTCs detection must also be established. Then, clinical research into CTCs as a circulating marker needs to be performed, and issues and promising results should be validated in large-scale, long-term follow-up, prospective clinical trials to ensure clinical applicability. Furthermore, conducting more basic research to gain an in-depth understanding of cancer biology may provide new insights into how and when to perform CTCs detection with the best clinical use. Despite these obstacles, we still have enough reason to believe that, with advances in detection and subsequent analytical techniques, CTCs will provide abundant useful information for the diagnosis and therapy in clinical practice for patients with GI cancers in the near future.

## Author Contributions

BX designed the review. CY and FC drafted the manuscript and prepared the figures. SW helped to modify the manuscript. All authors read and approved the final manuscript.

### Conflict of Interest

The authors declare that the research was conducted in the absence of any commercial or financial relationships that could be construed as a potential conflict of interest.
